# Personal Perspective on Understanding Low Molecular Weight Gels

**DOI:** 10.1021/jacs.2c02096

**Published:** 2022-06-17

**Authors:** Dave J. Adams

**Affiliations:** School of Chemistry, University of Glasgow, Glasgow G12 8QQ, United Kingdom

## Abstract

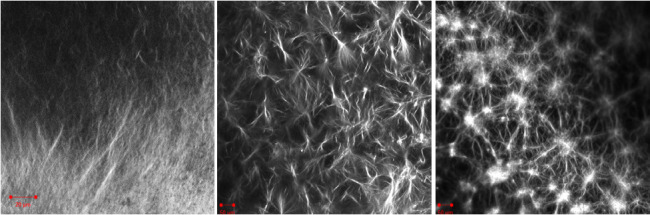

Low molecular weight gels are formed by the self-assembly of small molecules into anisotropic structures that form a network capable of immobilizing the solvent. Such gels are common, with a huge number of different examples existing, and they have many applications. However, there are still significant gaps in our understanding of these systems and challenges that need to be addressed if we are to be able to fully design such systems. Here, a number of these challenges are discussed.

Gels are formed from a matrix that immobilizes a liquid. The liquid is the majority component and can represent >99% of the total mass, but overall, the gel behaves as a solid. The properties of a gel are mainly a result of the matrix despite this being the minority component. Different types of matrices are possible, giving varying properties to the resulting solid-like material.

Gels can be prepared by polymerizing monomers and cross-linkers to form covalently cross-linking polymer chains.^1,2^ Parameters such as average polymer length, number of cross-links, type of cross-link (joining one, two, three, or more chains together, for example), and distances between the cross-links can be controlled by the chemistry used to form the matrix. Gels formed in this manner can have a range of stiffness and typically can be stretched, for example, to some degree, molded into different shapes with memory, dried, and resolvated. Such gels are however essentially permanent, and it is difficult to break them down as they are formed by covalent bonds.

It is also possible to form gels by cross-linking preformed polymer chains. Good examples are biopolymers such as alginates where the biopolymer backbone chemistry is rich in carboxylic acids.^3,4^ As such, deprotonation and placement in a calcium-rich environment results in chelation of calcium ions and hence cross-linking of the biopolymer chains; the gel properties can be controlled by the concentration of the biopolymer and cross-linking density. Similarly, gels can be formed by heating and cooling solutions of gelatin, which results in a reversible transition leading to solubilization (at high temperature) and helix formation on cooling, which leads to the formation of cross-links between the gelatin chains. These physical gels are reversible.

A further type of gel that I will focus on here is formed by so-called low molecular weight gelators (LMWG).^5−8^ These gels are formed by the self-assembly of the LMWG into one-dimensional supramolecular structures that form the chains that underpin the gel network (Figure 1). These chains are assumed to cross-link either by physically wrapping around one another, branching of growing chains, or forming insoluble structures that simply cannot flow past one another. The self-assembly into these one-dimensional objects can be triggered by a number of means that depend on the chemical structure of the LMWG, but a common method is to simply heat and cool. Heating results in dissolution of the LMWG, with cooling leading to the molecules becoming insoluble. If the kinetics of the process are right and if the molecule is designed effectively, this will lead to gelation as opposed to crystallization or precipitation. Another example is a pH trigger whereby the LMWG is soluble (or at least dispersible) at a certain pH but becomes insoluble when the pH is changed. Again, assuming the kinetics of the pH change and molecular design are right, gelation will occur.

**Figure 1:**
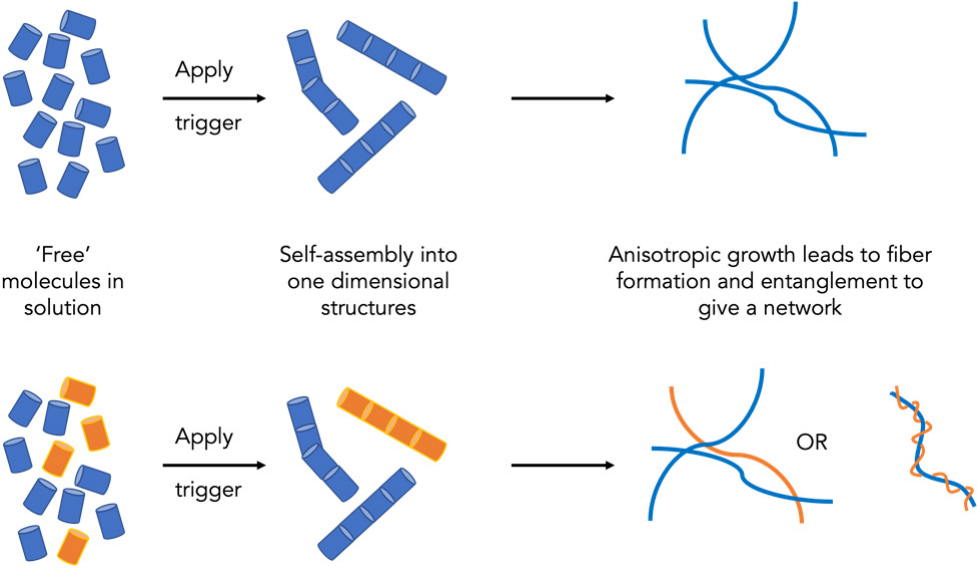
(a) Example cartoon description of a LMWG assembling to form fibrous structures that entangle to form a network. (b) In a two-component system, self-sorting can occur with both cartoon networks shown on the right being also described as self-sorted.

Such low molecular weight gels tend to be stiff (i.e., relatively high storage modulus, *G*′), the value of the loss modulus, *G*″, being around an order of magnitude lower (although this is very much not always the case, meaning that there can sometimes be discussions as to whether true gels are formed depending on one’s accepted definition of a gel), and tend to break at relatively low strain values. Hence, it is typically not possible to stretch such gels, for example. The gels can usually be converted easily back to the liquid phase by reversing the trigger (for example, reheating a gel formed by a heat–cool cycle will typically resolubilize the LMWG to give a solution). This may or may not be an advantage depending on the desired application but does mean that such gels are widely used in sensors, for example.

A major challenge in this field is that there is little understanding as to how to predict whether a molecule will be able to form a gel or instead form crystals or a precipitate. It is common to suggest design, but in most cases, gelators are found by accident or by synthetic permutation of a preexisting gelator. There have been suggestions that one can use crystal structures and crystal engineering to inform design.^9^ My personal feeling is that this is not terribly useful since this simply informs as to molecules that crystallize and hence not the gel phase. Indeed, we have recently shown conclusively that the crystal phase and the gel phase are different.^10^ The synthon approach^11,12^ suggests using specific functional groups to build in the necessary interactions, but this still essentially involves synthetic permutation of a preexisting gelator. There has been some recent progress in terms of understanding gelation in terms of solvent parameters,^13,14^ but this does not seem to allow prediction of what molecules will form a gel but more an understanding as to what solvents a particular gelator will gel. More successfully, there has been progress using computational approaches to predict gelation ability.^15−17^ To date, however, there is limited work showing how gel *properties* can be predicted, which is a major opportunity.

In this perspective, I intend to discuss some recent thoughts and observations arising from the 14 years with which I have worked with such systems. The aspects I will discuss are based around the challenges that would seem to need to be addressed if we are to truly be able to understand and design such systems. The first challenge is around language and the words used to describe the systems. The second challenge is how we characterize what has been formed across all length scales such that we can truly understand the properties of the gels. The third challenge is homogeneity, both in terms of preparing homogeneous gels reproducibly and in terms of homogeneity of structure and microstructure across the entire gel. The fourth challenge is understanding how the surface chemistry and confinement effects control and affect the properties of the gels. These aspects currently seem to me to be those which are perhaps the most difficult to control and understand but are critical if we are to be able to use these gels for the much-touted range of applications which include (but not exclusively) cell culturing,^18−20^ cancer therapy,^21,22^ drug delivery,^23,24^ optoelectronics,^25,26^ remediation,^27^ and sensing.^28^

Taking these challenges in turn, there are a number of descriptions that can cause confusion in my opinion. The gelators self-assemble into supramolecular structures including fibers, tapes, and tubes. Gelation is a result of entanglement or cross-linking of such structures, but it is not really appropriate to say that self-assembly leads to gelation but rather self-assembly leads to the formation of structures that, under appropriate conditions, interact to form a network that results in a gel being formed. It is entirely possible for self-assembly to occur but no gel being formed if the concentration is below the critical gelation concentration, for example. Importantly, in describing the self-assembled structures themselves, some of this language can cross from fields such as that of amyloid misfolding, and it is worth highlighting that different words may be used to describe what are very similar self-assembled structures. In terms of the network, cross-linking is often invoked. It is an interesting question as to whether this implies specific interactions or points where two fibers are bound together in some way or whether entanglement is also covered in this term. In many cases, it is not clear if there are formal cross-links or whether the network is essentially formed by long, insoluble objects jamming against one another.^29^

Linked to this is the description of structures as fibers (for example). In many cases, in aqueous situations, it is perhaps better to think of the structures formed as wormlike micelles, at least under certain situations. There are interesting discussions available as to gels formed from fibers and wormlike micelles,^29^ but when charged structures are formed, the latter description may be more appropriate.

For the network, there are length scale issues that are often not discussed. Most cartoons used to describe systems show the primary structures being formed. Likewise, there is a tendency to examine such gels using transmission electron microscopy (TEM), focusing on the nanostructures formed. However, the network that leads to gel formation will be on a much longer length scale, and so to understand the gel, it is necessary to examine the microstructure and macrostructure. I highlight this here as the descriptions on the nanostructures can dominate and are often used to discuss or even explain the gel properties, which likely misses out many length scales.

There is then the use of the term “gel”. It is common to claim a gel on the basis of a lack of flow in an upturned vial. However, for a number of systems, full rheological characterization can show that a gel is not formed.^30^ It is also not uncommon to see a claim of a frequency-independent material where the data clearly show that this is not the case or even for people to show only a very small frequency range! Exactly what is a gel can be difficult to define, but a good rule of thumb is that the frequency sweep shows a lack of frequency dependence and *G*′ is around an order of magnitude greater than *G*″.

When we move to multicomponent systems, the descriptions become even more difficult. As one example, self-sorted systems are very interesting where two gelators independently self-assemble into structures which only contain one of the gelators (Figure 1b).^31,32^ However, once these primary structures are formed, these can then conceptually go on to form interactions with structures either from the same gelator or from the other gelator (Figure 1c). Both of the cartoon structures formed in Figure 1c would currently be described as self-sorted but are clearly very different. Currently, there is no language available that captures this complexity, and most examples of self-sorted systems essentially show the cartoon of the primary structures, not the network. There is significant work needed here both to determine what is formed (a challenge in itself) and to find the best way of describing it.

Moving to the second challenge, it is a simple statement to make that the gel properties are controlled by the matrix, but this statement directly leads to a couple of significant questions. First, how does one know *what* matrix has been formed (for example, what types of cross-links are there, what is the cross-link density, how homogeneous is the network)? Second, can the matrix be controllably adjusted to give gels with specific properties or are we always postrationalizing these materials?

From this perspective, it is common in the field of LMWG to rely on cartoons to explain and describe the gels (Figure 1). Such cartoons often implicitly convey the idea of homogeneity of self-assembled structures, perhaps homogeneity of the cross-link density, and certainly homogeneity of the type of network. Rheological data where provided tend to be bulk rheology data with comparisons being made between gels on the basis of *G*′ and *G*″. While useful, in our experience all of this is probably oversimplistic. Such cartoons also usually imply molecular dissolution of the LMWG prior to gelation being triggered. This may not always be the case (especially for many water-based systems).

To provide specific examples of these points, gels can be formed in different ways from the same LMWG (Figure 2a).^33^ We have shown that 2NapFF can form gels via a pH switch, being dispersed at high pH, forming surfactant-like structures depending on the concentration and pH. At 5 mg/mL, hollow tubes are formed. When the pH is decreased, a structural transition to fibers occurs and a gel is formed.

**Figure 2:**
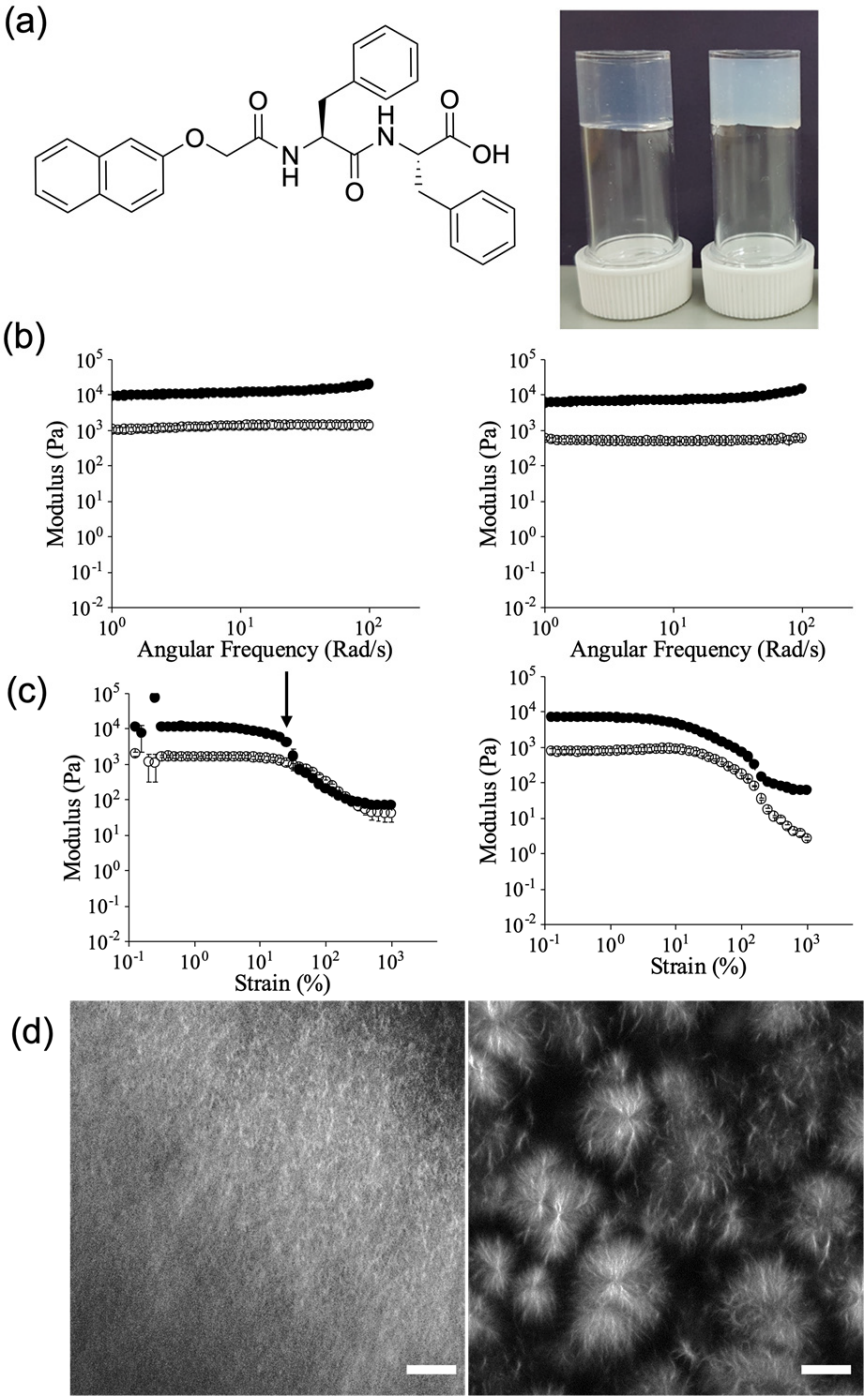
(a) Chemical structure of 2NapFF and gels formed from 2NapFF by (left) a pH switch and (right) a solvent trigger. (b) Example frequency sweeps for gels formed from 2NapFF at a concentration of 5 mg/mL by (left) a pH switch and (right) a solvent switch. (c) Example strain sweeps for gels formed from 2NapFF at a concentration of 5 mg/mL by (left) a pH switch and (right) a solvent switch. (d) Example confocal microscopy images showing differences in microstructure underpinning the gels formed from 2NapFF by (left) a pH switch and (right) and a solvent trigger. Data in a and d are adapted with permission from ref (49). Copyright 2019 Royal Society of Chemistry https://creativecommons.org/licenses/by/3.0/. The data in b and c are replotted from data in ref (33).

Gels can also be formed using a solvent switch whereby 2NapFF is initially dissolved in DMSO at a high concentration. When water is added, gelation occurs. In both of these cases, the rheological data implies that for the frequency sweeps the gels are very similar.^33^ The absolute values of *G*′ and *G*″ are similar and in all cases are largely independent of frequency (Figure 2b). However, there are some significant differences in the strain sweeps (Figure 2b). The gels formed by the pH switch break sharply at low strain (the breakage strain is shown by the arrow, and it can be seen that *G*′ decreases dramatically at this strain, dropping below *G*″). In contrast, the solvent-switch gels break at higher strain (shown by a gradual decrease in *G*′ and *G*″) and show evidence of creaming whereby there is never a crossover of *G*′ and *G*″. Hence, were one to only examine the frequency sweeps, one might draw the conclusions that these gels are similar, but the strain sweeps show that they are not.

A key issue in many cases is how does one analyze such gels effectively. The concentration of LMWG tends to be very low (<1 wt % in many cases, with gels reported where the LMWG concentration is <0.01 wt %),^5^ which can mean that some techniques are more appropriate than others. There is still a tendency to report new LMWG or libraries of LMWG and focus heavily on molecular packing. It is not clear to me that this adds much information to understanding the mechanical properties of the gel, and there are interesting questions as to what number of molecules different experimental techniques report over. Molecular packing is typically determined by spectroscopic means. For example, if H aggregation is reported or the formation of aggregates from fluorescence data, exactly how many molecules are packed in such a manner? How homogeneous are the structures formed? These are interesting points but difficult to answer.

A more important point for the discussion here in terms of understanding the matrix is into what structures are the LMWG self-assembled. A range of structures has been reported from fibrils and fibers, nanotubes, tapes, and helical structures. These are of course determined by how the molecule is packing, and it is not uncommon to use the dimensions of the LMWG to understand the packing within these structures. The absolute structures formed are typically determined by microscopy or scattering. There are many suitable microscopy techniques that can be used. A common issue is the use of techniques that require drying of the gels.^34^ Drying leads to concentration of the LMWG and can easily lead to changes in morphology and, in some cases, crystallization. Artifacts are extremely common with such techniques,^34^ especially when one adds a stain to aid with the imaging. Cryo-TEM is very effective and does not require drying, but it is hard to image the gel network since there is a maximum thickness for this technique.^35,36^ Hence, one often sees “free” structures as opposed to the network, and it is not uncommon to see very aligned structures, for example, which is presumably an artifact of the shear forces induced during the blotting process. Confocal microscopy can be really useful in terms of accessing the microstructure of the gels.^35,37^ Super-resolution microscopy techniques can also be used effectively.^35,38^ However, for both confocal and super-resolution microscopy, addition of a dye is normally required which can lead to questions as to whether the addition of the dye (or the synthesis of a dye-functionalized LMWG) has affected the outcome of the self-assembly. There are examples where the addition of a supposedly innocent dye leads to changes in the self-assembly. Small-angle scattering is an effective tool in comparison,^39−41^ allowing data to be collected in the gel state without drying, and provides information on average of the bulk sample. It has been found that both small-angle X-ray scattering (SAXS) and small-angle neutron scattering (SANS) and indeed a combination exploiting contrast matching approaches are extremely useful tools to understand our gels. These techniques are perhaps not as available to the average gel scientist, requiring an understanding of data fitting to models as well as often access to large-scale facilities. It can also be necessary to access other data to inform models. However, despite these caveats, SAXS and SANS are underused in this field, and I really hope to see more use in the near future (even if it is common for referees to still request microscopy!). The perceived lack of accessibility can be overcome by collaboration if necessary, and I believe it will be more useful long term than the focus on techniques that have the drawbacks mentioned above.

Returning to the example of the 2NapFF gels, SANS data showed that the underlying fibrous structures were different for all three gels.^33^ The data for the pH-triggered gel could be fitted to an elliptical cylinder model; we later showed that the elliptical cylinders could be interpreted from the perspective of lateral association of cylindrical structures.^42^ The data for the solvent-switch gels could not be fit to a model capturing a specific cross-sectional shape but rather to a Guinier–Porod model with the fit implying therefore that the scattering is from a relatively smooth surface formed by rods. All of this shows that the gels are underpinned by different self-assembled structures despite in both cases being formed from the same LMWG at the same pH and concentration.

Correlating with the SANS data and perhaps the most useful technique in this case to understand the differences in these systems is confocal microscopy, which showed that there are significant differences in the microstructure (Figure 2d).^33^ The pH-triggered gels show that there is a uniform distribution of fibers with perhaps the calcium-triggered gels showing more evidence of a tendency to be aligned. The solvent-switch gels however show spherulitic domains of fibers. This arises from a nucleation and growth event that occurs when water is added to the DMSO solutions of the LMWG. This explains the differences in the strain sweeps. The pH-triggered gels break sharply at low strain because the network is formed by rigid fibers that laterally associate; presumably in this case, the fibers are not strongly cross-linked, and so the network can be thought of as rigid rods being pushed against one another that, like uncooked spaghetti, can withstand a certain strain before breaking. With this analogy, it is therefore not surprising that these gels do not recover their rheological properties after the application of a high strain. The network is broken, and there is no way to recover this without a full reversal to the sol state and regelation. The solvent-switch gels show creaming in the strain sweeps as the spherulitic domains are essentially spheres of fibers jammed against each other which flow when strain is applied. These gels recover their rheological properties well after high strain is applied as rejamming occurs. Similar observations for such a microstructure were also reported by the groups of Schneider and Pochan.^43^ As a result, we have shown that the solvent-triggered gels can be 3D printed effectively, while the pH-triggered gels cannot (Figure 3).^44^ This ability to be 3D printed arises from the microstructure, not from the molecular structure, the molecular packing, or the primary fibers.

**Figure 3:**
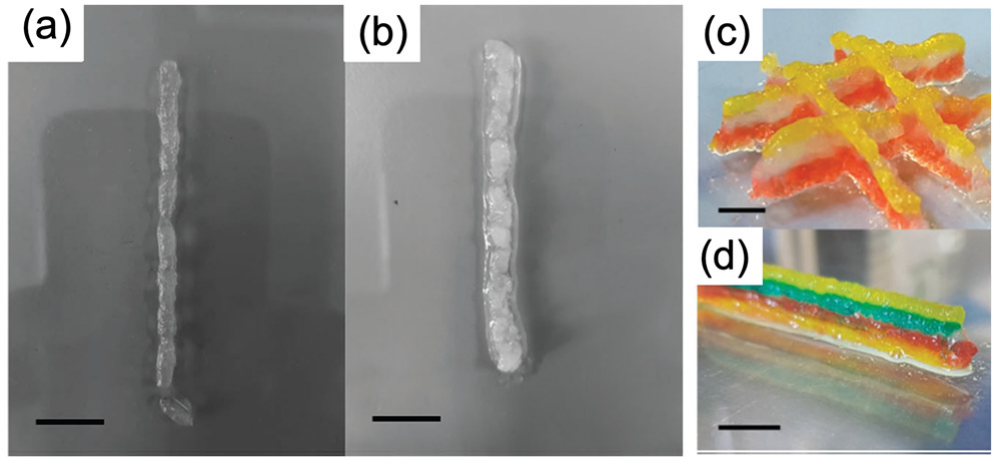
Gel microstructure affects bulk properties. (a) Gel formed by a specific LMWG by a solvent-triggered approach forms a spherulitic microstructure which means the gel can be directly 3D printed. (b) In comparison, a gel formed from the same LMWG by a pH-triggered approach forms a different microstructure and so cannot be successfully 3D printed. (c and d) Example structures that can be directly 3D printed from such gels as long as the microstructure is correct. Images are adapted with permission from ref (44). Copyright 2019 Royal Society of Chemistry https://creativecommons.org/licenses/by/3.0/.

Returning to the question as to how one knows what matrix has been formed, the above shows that we can understand key aspects using a combination of small-angle scattering and microscopy. The small-angle scattering data can allow us to determine the primary fibers (or other self-assembled aggregates) and can, to some degree, allow us to understand how homogeneous these structures are. In some cases, good fits to the data can be obtained from specific models. However, in other cases, it is necessary to either build in polydispersity in a parameter such as the radius or to mix models. The inclusion of polydispersity implies that the structures are not homogeneous, while the need for multiple models shows that different populations exist. However, it is difficult to gain much information from such scattering as to the cross-link type or density in many cases. In several of our samples, we see excess scattering at low *Q* (long length scales), which we take into account by adding a power law to the fit. This seems to be scattering from the network but does not help with understanding the cross-linking.

Microscopy can be used to show how uniform the microstructure is across the gel. Multiple images can be taken to understand how similar or not the sample is at different points. Again, it is difficult to quantify or understand cross-linking. Some examples exist where specific types of cross-links have been assigned on the basis of microscopy data,^45^ for example, fiber branching.^46^ However, such examples usually show a small section of the gel, so they do not necessarily show that all cross-linking is the same. Again, we highlight that there may well be drying artifacts arising. From this perspective, the lack of being able to determine the type and concentration of the cross-links represents a significant issue in understanding such gels. The gel properties result from the network, but it is extremely difficult to access all of the necessary information about this network. As discussed above, there are indications as to different types of networks and microstructures from the strain sweeps shown in Figure 2, but there is a need for significantly more work and insight here.

The third challenge, homogeneity, is an interesting point from the perspective of rheological analysis. Rheology on a sample will provide data even if the gel is very heterogeneous. Some understanding of heterogeneity or irreproducibility in sample preparation can be accessed by repeat measurements on fresh gels, but if each gel is heterogeneous in properties, this may not be determined from bulk rheological measurements. It is possible to probe more locally using cavitation rheology^47^ or nanoindentation,^48^ and these data can show whether a gel is uniform or not. However, typically nanoindentation can only probe the surface of the gel (which may not be the same as the bulk). Cavitation rheology is an interesting alternative by which a bubble (for example of air) is pumped into a gel and the pressure monitored as the bubble size increases.^47,49^ The maximum pressure that can be withstood can be used to compare different gels, and importantly, it is possible to use this approach to probe multiple points within the same gel and therefore determine whether the mechanical properties of a gel are homogeneous (at least on the length scale of such a bubble). A number of other rheological experiments are also possible.^50^

The final challenge is around interesting aspects that are becoming clearer in terms of their effect on gels including the impact of the container in which the gels are formed. The surface chemistry of the vial can affect the outcome of gelation and the properties of the resulting gels.^51^ Less discussed aspects are the size and shape of the vials. Gels can be formed from LMWG in all shapes and sizes as typically there is sufficient time to transfer the solution after the trigger has been applied before gelation. Alternatively, a trigger can be added to a solution in different shape vials. This means that gels have been prepared in different interesting shapes and also in useful volumes and geometries. It is not always possible to measure properties of the resulting gels due to the sample size, and it is common to measure the rheological properties of the “bulk” gel and assume the properties translate to the smaller volumes or different shapes. For example, when using gels in well plates for cell culturing, the dimensions typically are too small for rheology to be carried out. It is not clear however that one can extrapolate across length scales.

To start with, the absolute surface to volume ratio will change, and depending on whether and how each interface affects the outcome of the self-assembly, there could be significant differences in the morphology and microstructure. Second, if the microstructure cannot change, constraining this within different dimensions opens questions as to how the network is arranged within different environments. As a single example, there are a number of suggestions of using such LWMG to form gels within emulsion droplets,^52^ thin films,^53^ confined spaces,^54^ interfacial gels,^55,56^ compartmentalized gels,^57^ and patterned surfaces.^58^ By default, this constrains the dimensions of the object in which the assembly occurs. It seems unlikely that there can be no changes in the self-assembled structure, cross-links, and microstructure, and hence, it is an open question as to how much can be learned about such systems when using information collected in the bulk. It also seems likely that the type of microstructure formed must be affected differently. Thus, how gelation is carried out will be an important aspect.

Other points of consideration from the point of view of the dimensions of the sample container are the kinetics of assembly. Many of these gels are very much under kinetic control, with the properties of the gels depending on the rate of gelation, mixing, in addition to several other experimental aspects. These parameters can be difficult to control, and this will be exacerbated when trying to compare across gels formed in different types of containers. For example, gels formed by a heat–cool method in large glass vials versus in wells in a 96-well plate are unlikely to cool at the same rate and therefore unlikely to have the same properties. Hence, there are interesting questions as to how much differences in gels formed in such different containers will be driven by confinement effects and how much will be driven by changes in kinetics.

Overall, these gels are interesting, and it is very clear that they can be used for a wide range of applications. However, despite being around for many years, there are still significant gaps in our understanding. My group’s experience is that much of the literature is hard to reproduce. In some cases, this is due to protocols being described insufficiently clearly as it turns out that there are extremely important subtleties to the gel formation process that must be followed; these are not always clear at the time of publication and so may be missed out of the methodology. There are gels now, for example, where the stirring rate during stock solution preparation is critical, an aspect which has not been noticed previously for these samples. Couple such issues with a lack of techniques to probe effectively cross-links, a tendency to report limited rheology data, and many examples where data is shown that may well be suffering from drying artifacts and we have systems which are really interesting but far from being well described. Further issues such as surface chemistry effects and confinement effects provide interesting challenges as well as making it sometimes difficult to reproduce work. Overall, these challenges are extremely interesting (at least to me!) and provide real opportunities. As but one example, it has recently been shown that changing the underlying surface on which a gel is formed results in changes in the properties of the gels grown on these surfaces.^59^ It is therefore possible to prepare gels with patterned properties by growing them on patterned surfaces. It is difficult to imagine how this could be achieved in so simple a manner. Hopefully in not too long the challenges I have discussed here will be addressed and we will be at a point where the seemingly ubiquitous statement that these gels are hard to design will no longer be needed in the introduction to papers!
